# Frequency-Dependent Block of Excitatory Neurotransmission by Isoflurane via Dual Presynaptic Mechanisms

**DOI:** 10.1523/JNEUROSCI.2946-19.2020

**Published:** 2020-05-20

**Authors:** Han-Ying Wang, Kohgaku Eguchi, Takayuki Yamashita, Tomoyuki Takahashi

**Affiliations:** ^1^Cellular and Molecular Synaptic Function Unit, Okinawa Institute of Science and Technology Graduate University, Okinawa, 904-0495, Japan; ^2^Molecular Neuroscience Group, Institute of Science and Technology Austria, Klosterneuburg, 3400, Austria; ^3^Department of Neuroscience II, Research Institute of Environmental Medicine, Nagoya University, Nagoya, 464-8601, Japan

**Keywords:** calyx of Held, cerebral cortical synapse, exocytic capacitance change, frequency-dependent inhibition, isoflurane, presynaptic Ca^2+^ currents

## Abstract

Volatile anesthetics are widely used for surgery, but neuronal mechanisms of anesthesia remain unidentified. At the calyx of Held in brainstem slices from rats of either sex, isoflurane at clinical doses attenuated EPSCs by decreasing the release probability and the number of readily releasable vesicles. In presynaptic recordings of Ca^2+^ currents and exocytic capacitance changes, isoflurane attenuated exocytosis by inhibiting Ca^2+^ currents evoked by a short presynaptic depolarization, whereas it inhibited exocytosis evoked by a prolonged depolarization via directly blocking exocytic machinery downstream of Ca^2+^ influx. Since the length of presynaptic depolarization can simulate the frequency of synaptic inputs, isoflurane anesthesia is likely mediated by distinct dual mechanisms, depending on input frequencies. In simultaneous presynaptic and postsynaptic action potential recordings, isoflurane impaired the fidelity of repetitive spike transmission, more strongly at higher frequencies. Furthermore, in the cerebrum of adult mice, isoflurane inhibited monosynaptic corticocortical spike transmission, preferentially at a higher frequency. We conclude that dual presynaptic mechanisms operate for the anesthetic action of isoflurane, of which direct inhibition of exocytic machinery plays a low-pass filtering role in spike transmission at central excitatory synapses.

**SIGNIFICANCE STATEMENT** Synaptic mechanisms of general anesthesia remain unidentified. In rat brainstem slices, isoflurane inhibits excitatory transmitter release by blocking presynaptic Ca^2+^ channels and exocytic machinery, with the latter mechanism predominating in its inhibitory effect on high-frequency transmission. Both in slice and *in vivo*, isoflurane preferentially inhibits spike transmission induced by high-frequency presynaptic inputs. This low-pass filtering action of isoflurane likely plays a significant role in general anesthesia.

## Introduction

Volatile anesthetics have been widely used for surgery since the 19th century. Sherrington (1906) predicted that the synapse is the main target of volatile anesthetics. At inhibitory synapses, volatile anesthetics prolong postsynaptic responses (Nicoll, 1972; Mody et al., 1991); and at both excitatory and inhibitory synapses, they inhibit neurotransmitter release (Takenoshita and Takahashi, 1987; Kullmann et al., 1989; Wu et al., 2004; Baumgart et al., 2015). Various mechanisms have been postulated to explain presynaptic inhibitory effects of volatile anesthetics. These include (1) inhibition of voltage-gated Na^+^ channels (Haydon and Urban, 1983; Rehberg et al., 1996; Ouyang and Hemmings, 2005); (2) inhibition of voltage-gated Ca^2+^ channels (Study, 1994; Kamatchi et al., 1999); and (3) activation of voltage-independent K^+^ channels (Patel et al., 1999; Ries and Puil, 1999; Franks and Honore, 2004). Volatile anesthetics are also proposed to (4) directly block vesicle exocytosis via inhibiting vesicle fusion machineries (van Swinderen et al., 1999; Nagele et al., 2005; Herring et al., 2009; Xie et al., 2013). However, the primary target of anesthetics remains unidentified.

Recently, at hippocampal synapses in culture, Baumgart et al. (2015) reported that isoflurane inhibits presynaptic Ca^2+^ influx without changing the Ca^2+^-release relationship. Hence, they postulated that a reduction of Ca^2+^ influx fully explains presynaptic inhibitory effect of isoflurane. Since this conclusion is based on experiments using single action potential (AP) stimulation, the target of the anesthetics on repetitive neurotransmission remains open. It also remains unidentified whether the reduction of Ca^2+^ influx is caused by direct inhibition of Ca^2+^ channels or indirectly caused by a reduction of presynaptic AP amplitude. At the calyx of Held in brainstem slices from prehearing rats, Wu et al. (2004) did not observe consistent inhibition of presynaptic Ca^2+^ channel currents by isoflurane but found that isoflurane reduces presynaptic AP amplitude, thereby proposing that the latter mechanism may mediate inhibition of transmitter release by isoflurane.

Hence, using the calyx of Held in posthearing rat brainstem slices, we systematically addressed the target of isoflurane. In variance-mean analysis, isoflurane attenuated both the release probability (*p*_r_) and the number of functional release sites (*N*), suggesting that multiple mechanisms likely underlie the isoflurane effect. In presynaptic recordings, we consistently found that isoflurane inhibited voltage-gated Ca^2+^ channels (VGCCs) ofP/Q type, thereby reducing Ca^2+^ influx. Isoflurane also inhibited voltage-gated Na^+^ channels and reduced presynaptic AP amplitude; but in contrast to previous proposal (Wu et al., 2004), this effect could not explain the reduction of EPSC amplitude by isoflurane because of the wide safety margin of AP amplitude for transmitter release (Hori and Takahashi, 2009). When vesicle exocytosis was triggered by Ca^2+^ through VGCCs activated by a short depolarizing pulse, isoflurane inhibited exocytosis via inhibiting Ca^2+^ influx. However, when more massive exocytosis was induced by a long presynaptic depolarization, isoflurane directly inhibited exocytic machinery downstream of Ca^2+^ influx. In simultaneous recordings of presynaptic and postsynaptic APs, isoflurane preferentially impaired the fidelity of transmission at higher frequencies. Likewise, in unit recordings from cerebral cortical neurons in mice *in vivo*, isoflurane preferentially inhibited monosynaptic transmission evoked by a higher-frequency stimulation. Thus, isoflurane inhibits excitatory transmission by dual mechanisms, of which its direct inhibitory effect on exocytic machinery significantly contributes to general anesthesia by low-pass filtering excitatory spike transmission.

## Materials and Methods

All animal experiments were performed in accordance with guidelines of the Physiologic Society of Japan and institutional regulations of animal experiments at Okinawa Institute of Science and Technology and Nagoya University Research Institute of Environmental Medicine.

### 

#### Slice preparation and solutions

Wistar rats (postnatal day [P] 13–15) of either sex were killed by decapitation under isoflurane anesthesia. Transverse brainstem slices (175–200 μm in thickness) containing the medial nucleus of the trapezoid body (MNTB) were cut in ice-cold solution containing the following (in mM): 200 sucrose, 2.5 KCl, 26 NaHCO_3_, 1.25 NaH_2_PO_4_, 6 MgCl_2_, 10 glucose, 3 *myo*-inositol, 2 sodium pyruvate, and 0.5 sodium ascorbate (pH 7.4 when bubbled with 95% O_2_ and 5% CO_2_, 310-320 mOsm) by using vibroslicer (VT1200S, Leica Microsystems). Before recording, slices were incubated for 1 h at 36°C–37°C in standard aCSF containing the following (in mM): 125 NaCl, 2.5 KCl, 26 NaHCO_3_, 1.25 NaH_2_PO_4_, 2 CaCl_2_, 1 MgCl_2_, 10 glucose, 3 *myo*-inositol, 2 sodium pyruvate, and 0.5 sodium ascorbate (pH 7.4 when bubbled with 95% O_2_ and 5% CO_2_, 310-320 mOsm), and maintained thereafter at room temperature (24°C–26°C). For recordings, aCSF routinely contained bicuculline methiodide (10 μM) and strychnine hydrochloride (0.5 μM) to block inhibitory synaptic transmission, unless otherwise mentioned. TTX (1 μM) was add to aCSF for recording mEPSCs. For postsynaptic EPSC recordings, pipette solution contained the following (mM): 110 CsF, 30 CsCl, 10 HEPES, 5 EGTA, 1 MgCl_2_, and 5 QX-314-Cl (pH adjusted to 7.3–7.4 with CsOH, 300-320 mOsm). For recording postsynaptic APs, pipettes contained the following (mM): 120 potassium gluconate, 30 KCl, 5 EGTA, 12 disodium phosphocreatine, 1 MgCl_2_, 3 Mg-ATP, 0.3 Na_2_-GTP, 1 l-arginine (pH 7.3–7.4 adjusted with KOH, 315–320 mOsm). For presynaptic K^+^ current recording, TTX (1 μM) was added to aCSF. For recording presynaptic Ca^2+^ currents or membrane capacitance, NaCl in the aCSF was replaced with tetraethylammonium chloride (TEA-Cl, 10 mM), and TTX (1 μM) and 4-aminopyridine (4-AP, 0.5 mM) were added. For presynaptic Na^+^ current recording, the extracellular Na^+^ concentration was reduced to 5%, being replaced by TEA-Cl (119 mM), to optimize voltage-clamp control. 4-AP (0.5 mM) and CdCl_2_ (200 μM) were added to aCSF to block K^+^ and Ca^2+^ conductance, respectively. In most experiments, presynaptic pipette solutions contained the following (mM): 105 cesium gluconate, 30 CsCl, 10 HEPES, 0.5 EGTA, 1 MgCl_2_, 12 disodium phosphocreatine, 3 Mg-ATP, 0.3 Na-GTP (pH 7.3–7.4 adjusted with CsOH, 315–320 mOsm). For presynaptic AP recording, potassium gluconate concentration was reduced to 110 mM from the postsynaptic pipette solution and 10 mM l-glutamate was supplemented. In experiments for testing the effect of presynaptic AP amplitude on EPSCs (see Fig. 3*E*), we added kynurenic acid (1 mM) to aCSF to minimize saturation of postsynaptic AMPA receptors (Koike-Tani et al., 2008).

#### Slice experiments

Recordings from slices were made mostly at room temperature (24°C–26°C), but AP recordings from presynaptic terminals and postsynaptic MNTB neurons were made at near physiological temperature (PT, 31°C–33°C). Simultaneous presynaptic and postsynaptic AP recordings (see Fig. 7) were performed at PT to improve synaptic fidelity (Kushmerick et al., 2006; Piriya Ananda Babu et al., 2020). MNTB principal neurons and calyx of Held presynaptic terminals were visually identified using a 40× water immersion objective (Olympus) attached to an upright microscope (BX51WI, Olympus). Whole-cell recordings were made from MNTB principal neurons and presynaptic terminals using an EPC-10 patch-clamp amplifier controlled by PatchMaster software (HEKA) after online low-pass filtering at 5 kHz and digitizing at 50 kHz. EPSCs were evoked by stimulation using a bipolar tungsten electrode (FHC) positioned between the midline and the MNTB region. MNTB neurons were voltage-clamped at a holding potential of −70 mV. The postsynaptic pipette was pulled to a resistance of 2-3 MΩ and had a series resistance of 4-10 MΩ, which was compensated by 40%-70% for a final value of 3 MΩ. For variance-mean analysis (Clements and Silver, 2000), EPSCs were evoked at 0.05 Hz in the presence of kynurenic acid (1 mM) under aCSF with various extracellular [Ca^2+^]/[Mg^2+^] (see Fig. 2). Fifteen successive EPSCs were collected for constructing a variance-mean plot. To acquire EPSCs at high release probability (*p_r_*), 4-AP (10 μM) was added to the aCSF (Koike-Tani et al., 2008). Plots of variance as a function of mean were fit by using the simple parabola equation as follows:

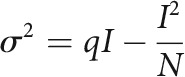
 where σ*^2^* and *I* represent the variance and mean amplitude of EPSCs, respectively. The parameters *q* and *N* denote the mean quantal size and the number of release sites, respectively. *q* can be estimated from the initial slope of the parabola. *Nq* can be estimated from the *X* intercept of the parabola, *p_r_* at 2 mM Ca^2+^ calculated as *I/Nq*.

For presynaptic recordings, pipettes were pulled at a resistance of 5-7 MΩ and had a series resistance of 9-25 MΩ, which was compensated by up to 80% for a final value of ∼7 MΩ. For measuring presynaptic Na^+^ and K^+^ currents, membrane potential was stepped up by 10 mV with a 20 ms pulse from −80 to 60 mV. For presynaptic Ca^2+^ current recordings, membrane potential was stepped up by 10 mV with a 20 ms pulse from −80 to 40 mV. The P/4 method was used for correcting leak and capacitance currents. For monitoring presynaptic membrane capacitance (C_m_; see Figs. 4, 5), pipette tips were coated with dental wax to reduce stray capacitance. A sinusoidal voltage command was applied with a peak-to-peak amplitude of 60 mV at 1 kHz. Samples of C_m_ were plotted as average values of 50 data points within 50 ms (short time scale) or 500 ms (long time scale). Presynaptic membrane capacitance changes (ΔC_m_) were induced by Ca^2+^ currents as described previously (Taschenberger et al., 2002; Sun et al., 2004; Yamashita et al., 2005, 2010). The amplitude of exocytic capacitance changes was measured between the baseline and the maximal value of C_m_ at 450-500 ms after depolarization to avoid contamination by artificial ΔC_m_ changes (Yamashita et al., 2005). For testing the fidelity of synaptic transmission, simultaneous presynaptic and postsynaptic whole-cell recordings in current-clamp mode were made at near PT.

**Figure 1.**
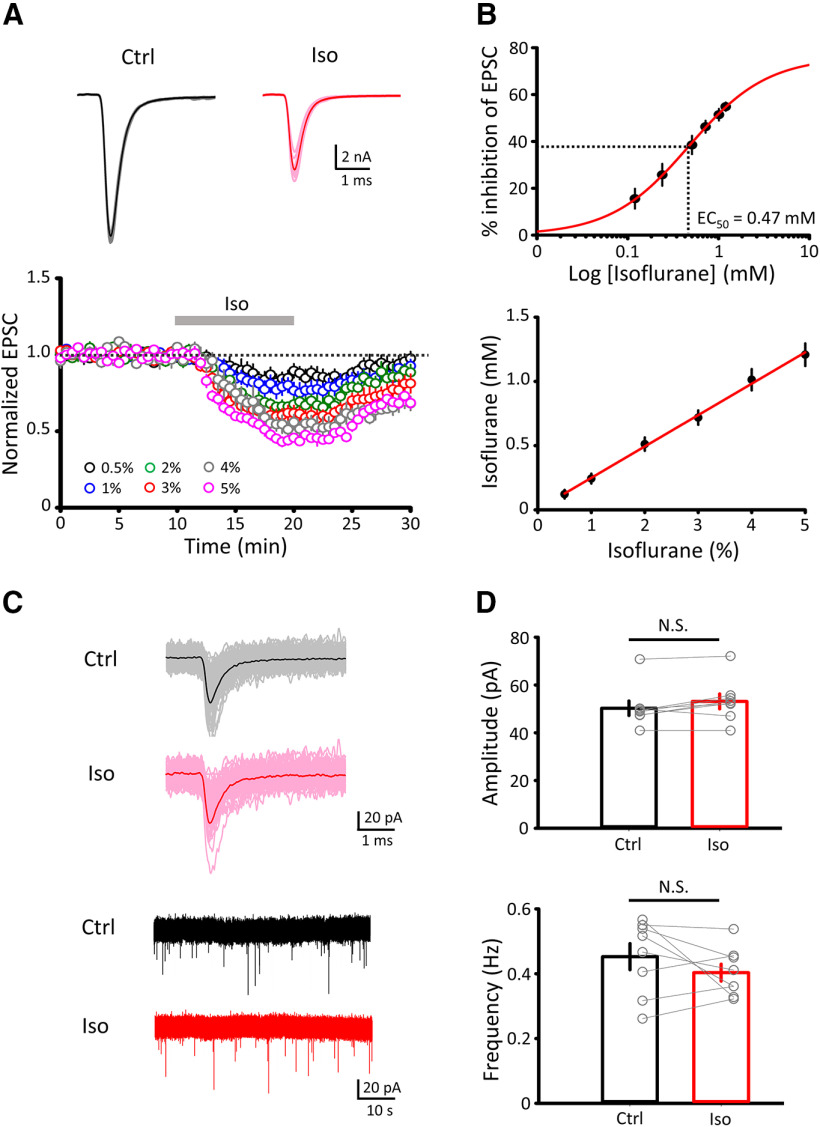
Isoflurane attenuates evoked EPSC amplitude. ***A***, Top, Sample records of EPSCs averaged from 6 cells each, before (Ctrl, black) and 10 min after 3% isoflurane application (Iso, red). Eight EPSCs are superimposed on averaged traces. Amplitudes of EPSC before and after 3% Iso application were 12.2 ± 1.4 nA and 5.6 ± 0.9 nA, respectively. Isoflurane did not affect the 10%-90% rise time (0.18 ± 0.01 ms in Ctrl, 0.18 ± 0.01 ms in Iso, *n* = 8, *p* = 0.63, paired-sample *t* test) or the decay time constant (0.41 ± 0.06 ms in Ctrl, 0.39 ± 0.06 ms in Iso, *n* = 8, *p* = 0.49, paired-sample *t* test) of EPSCs. Bottom, Time plots of EPSC amplitude attenuated by various gaseous concentrations of isoflurane. EPSCs were evoked every 30 s. After 10 min of baseline recording, isoflurane was bath-applied for 10 min. ***B***, Top, The fractional block of EPSCs by isoflurane at different isoflurane concentrations, fitted with a Hill equation: $Y=Max-\frac{Max}{1+{\left(\frac{X}{EC_{50}}\right)}^{Hill}}$, where the EC_50_ was 0.47 mM (dashed lines), maximal inhibition (Max) was 76%, and Hill coefficient (Hill) was 1.0. Bottom, A linear relationship between gaseous concentrations (%) and aqueous concentration (mM) of isoflurane measured using gas chromatography-mass spectrometry. ***C***, Top, mEPSCs averaged from 126 events each, before (Ctrl, black) and 10 min after isoflurane application (Iso, red). Bottom, Sample traces of mEPSCs at slow time scale. ***D***, Top, Isoflurane had no effect on mEPSC amplitude (50.3 ± 3.1 pA in Ctrl, 53.1 ± 3.2 pA in Iso, *n* = 8, *p* = 0.52, paired-sample *t* test). Individual (368) events are superimposed on averaged traces. Bottom, No effect of isoflurane on mEPSC frequency (0.45 ± 0.04 Hz in Ctrl, 0.40 ± 0.02 Hz in Iso, *n* = 8, *p* = 0.39, paired-sample *t* test). Error bars indicate ± SEM in this and subsequent figures. Ctrl, Control; Iso, isoflurane; N.S., no significance.

We applied isoflurane to slices as described previously (Wu et al., 2004). The gas mixture of 95% O_2_ and 5% CO_2_ was introduced by a flowmeter into acalibrated commercial vaporizer (MK-AT210, Muromachi) containing isoflurane (100%, 24.5 mM; see Fig. 1*A*). The gas mixture at various isoflurane concentrations (0.5%–5%) was then bubbled into the experimental solution in a tightly capped bottle. In most slice experiments, we used isoflurane at 3%, which was 0.72 ± 0.06 mM when examined using gas chromatography (see Fig. 1*B*) and corresponded to twice the minimum alveolar concentration (2 MAC) of isoflurane (Mazze et al., 1985; Pal et al., 2012). For testing neurotransmission fidelity (see Fig. 7), we applied isoflurane at 1.5% or 3% (1 or 2 MAC). The aqueous concentration of isoflurane, measured with gas chromatography-mass spectrometry (Pegasus 4D-C GCxGC-TOFMS, Saint Joseph), was linearlyproportional to the gaseous partial concentration of isoflurane (see Fig. 1*B*). For i*n vivo* experiments, isoflurane (1.4%) was applied by inhalation.

#### In vivo unit recordings

For optogenetic stimulation (see Fig. 8), we used double-transgenic mice expressing channelrhodposin 2 (ChR2) in layer 5 (L5) pyramidal neurons of cerebral cortex (*Rbp4 Cre; LSL-ChR2*) that were obtained by crossing Rbp4-Cre (Gensat STOCK Tg(Rbp4-cre)KL100Gsat/Mmucd) mice with Ai32 (Jax #012569). Adult *Rbp4-Cre; LSL-ChR2* mice were implanted with a lightweight metal head-holder and a recording chamber under isoflurane anesthesia, as previously described (Yamashita et al., 2013). A small craniotomy was opened over the left whisker primary somatosensory cortex (wS1, 1.65 mm posterior, 3.0 mm lateral from the bregma) (Yamashita et al., 2018) and left whisker primary motor cortex (wM1, 1 mm anterior, 1 mm lateral from bregma) (Sreenivasan et al., 2016). In some recordings, the location of the left wS1-C2 barrel column was also identified using intrinsic optical imaging under light isoflurane anesthesia (Ferezou et al., 2007; Yamashita et al., 2013). Extracellular spikes were recorded using a silicon probe (A1x32-Poly2-10mm50s-177, NeuroNexus) with 32 recording sites along a single shank, covering 775 mm of the cortical depth in awake states or under isoflurane (1.4%) anesthesia (Miyazaki et al., 2019). The probe was lowered gradually until the tip was positioned at a depth of ∼950 μm under the wS1 pial surface. Neural data werefiltered between 0.5 Hz and 7.5 kHzand amplified using a digital head-stage (RHD2132, Intan Technologies). The head-stage digitized the data with a sampling frequency of 30 kHz. The digitized signal was transferred to an acquisition board (Open Ephys) and stored on an internal HDD of the host PC for offline analysis. Photo-stimulation was conducted by applying 1 ms pluses of blue LED (19 mW) with an optical fiber (400 μm diameter) placed overthe wM1 craniotomy. Two sweeps of200 photo-stimuli at 0.2 Hz every 20 min were first applied in awake states; and subsequently, two sweeps of 0.2 Hz stimulation were applied under isoflurane anesthesia. Mice were then recovered by stopping isoflurane inhalation; and, after whisking behavior was observed as the mice awoke, five sweeps of 200 photo-stimuli at 2 Hz every 6 min were applied. Mice were again anesthetized by isoflurane inhalation and another five sweeps of 2 Hz stimulation were applied. Recordings with 0.2 Hz stimuli and 2 Hz stimuli were saved separately.

Spiking activity on each probe was detected and sorted into different clusters using Kilosort, an open source spikesorting software (https://github.com/cortex-lab/KiloSort). After an automated clustering step, clusters were manually sorted and refined. Only well-isolated single units (total 188 units) were included in the dataset. For analysis, we excluded units (34 of 188 units) that showed AP rates <5 Hz on average at 5–25 ms after photograph-stimulation in awake states. Units that have reliably evoked APs with a low jitter (∼1 ms) were tested for collisions in which we looked for absence of antidromic spikes when preceded by spontaneous spikes. Among these units, 11 units showed collisions between evoked and spontaneous APs (see Fig. 8*C*), indicating putative wS1-to-wM1 projection neurons generating antidromic spikes. These data were excluded from analysis. In total, 143 units (83 units for 0.2 Hz stimulation and 60 units for 2 Hz stimulation) were selected for analysis as trans-synaptically activated units. Averaged spontaneous AP rates were measured for a period of 50 ms before photo-stimulation onset at 0.2 Hz. Evoked AP rates were calculated by subtracting averaged AP rates 0–50 ms before photo-stimulation from those during 5–25 ms after photo-stimulation.

#### In vivo whole-cell recordings

Adult *Rbp4-Cre; LSL-ChR2* mice were implanted with a lightweight metal head-holder and a recording chamber under isoflurane anesthesia. After recovery, mice were habituated to head restraint (three sessions, one session per day) before recording. At the experimental day, a small craniotomy was opened over the left wS1 under isoflurane anesthesia. Recording pipettes (5–8 MΩ) were advanced into the cortex through the craniotomy with a positive pressure until the pipette resistance increased, and then suction was applied to establish a giga-ohm seal followed by the whole-cell configuration using a patch-clamp amplifier (Multiclamp 700B, Molecular Devices) (Margrie et al., 2002; Petersen et al., 2003). Pipettes were filled with a solution containing the following (in mM): 135 potassium gluconate, 4 KCl, 10 HEPES, 10 sodium phosphocreatine, 4 Mg-ATP, 0.3 Na_3_GTP (adjusted to pH 7.3 with KOH). Recordings were made at the putative subpial depth of 250–400 μm in a dark environment to avoid ChR2 activation. The membrane potential, which was not corrected for liquid junction potential, was filtered at 8 kHz and digitized at 20 kHz.

#### Data analysis

Data from slice experiments and *in vivo* whole-cell recordings were analyzed using IGOR Pro 6 (WaveMatrics), OriginPro 2015 (OriginLab), Sigmaplot 13 (Systat Software), and MS Excel 2016 (Microsoft). All values were expressed as mean ± SEM, and 95% CIs on the difference of the means were considered statistically significant in paired-sample *t* tests or one-way repeated-measures ANOVA with a *post hoc* Bonferroni test (*p* < 0.05), Mann–Whitney test (*p* < 0.05), or Kruskal–Wallis test with a *post hoc* Dunn's test (*p* < 0.05).

## Results

### Isoflurane attenuates EPSCs at the calyx of held

At the calyx of Held in slices, bath-application of isoflurane for 10 min attenuated the amplitude of EPSCs elicited in postsynaptic principal cells in the MNTB by input fiber stimulation (12.2 ± 1.4 nA in control, *n* = 8), in a concentration-dependent manner (Fig. 1*A*). The Hill plot fitted to the dose–response curve indicated that isoflurane inhibited EPSCs maximally by 76% with an EC_50_ of 1.9% that corresponds to 0.47 mM (Fig. 1*B*). Isoflurane had no effect on rise or decay time kinetics of EPSCs (for values, see Fig. 1 legend). In most following experiments, we used isoflurane at 0.72 mM (3%), which reduced EPSC amplitude by 46 ± 2.6% (Fig. 1*B*). This isoflurane concentration corresponds to 2 MAC for rats (Mazze et al., 1985; Taheri et al., 1991; Pal et al., 2012). Unlike neurally evoked EPSCs, isoflurane had no significant effect on the amplitude or frequency of spontaneous mEPSCs (Fig. 1*C*,*D*). Thus, these results are consistent with those reported at the calyx of Held in prehearing rat brainstem slices (Wu et al., 2004).

### Effects of isoflurane on quantal parameters

**Figure 2.**
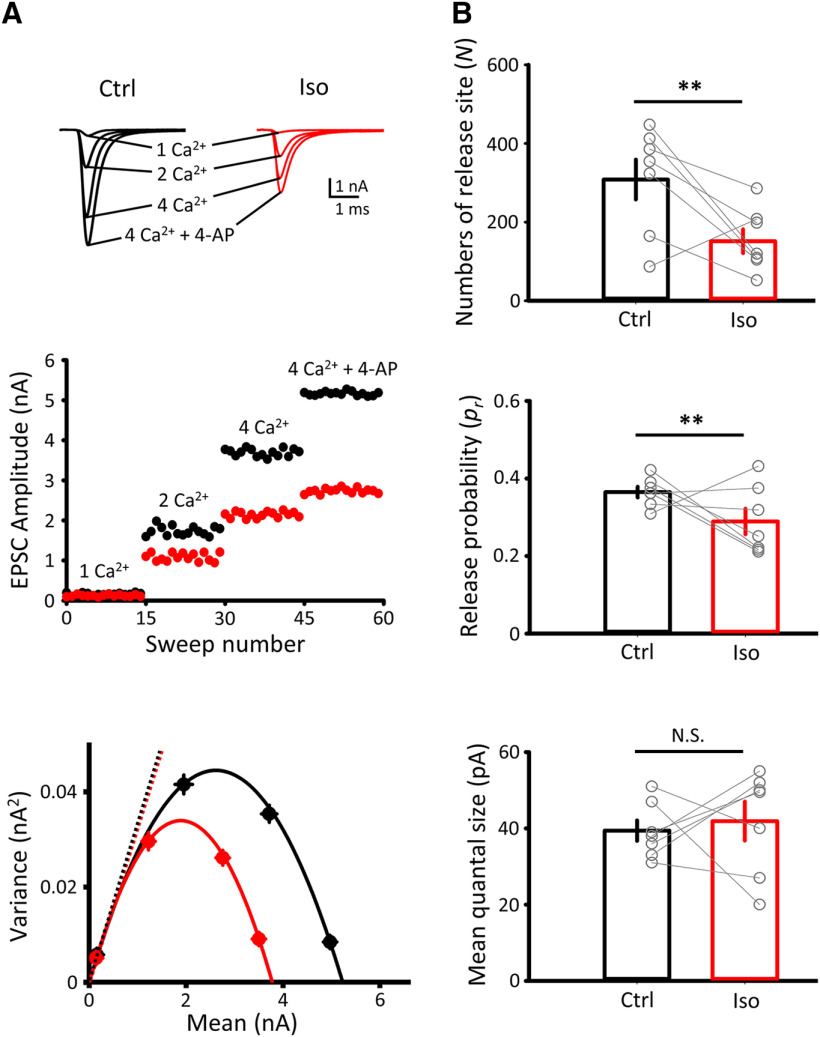
Isoflurane reduces the number of functional release sites (*N*) and the release probability (*p_r_*) but has no effect on mean quantal size (*q*). ***A***, Top, Sample traces of averaged EPSCs at different Ca^2+^ and Mg^2+^ concentrations superimposed with (red) or without (black) isoflurane (3%). Middle, EPSC amplitudes in aCSF solutions containing 1 mM Ca^2+^ and 2 mM Mg^2+^ (1Ca^2+^), 2 mM Ca^2+^ and 1 mM Mg^2+^ (2Ca^2+^), 4 mM Ca^2+^ and 0 mM Mg^2+^ (4Ca^2+^), and 4 mM Ca^2+^ and 0 mM Mg^2+^ with 10 μM 4-AP (4Ca^2+^ + 4-AP). aCSF contained kynurenic acid (1 mM) throughout to minimize saturation and desensitization of postsynaptic AMPA receptors. Bottom, Variance-mean plots in the absence (Ctrl, black) or presence of isoflurane (Iso, red, superimposed). Data points were fitted with simple parabolic functions (see Materials and Methods). ***B***, Bar graphs for the effect of isoflurane (3%) on *N* (top, 302 ± 45 in Ctrl, 171 ± 30 in 3% Iso, *n* = 7, *p* = 0.005, paired-sample *t* test), *p_r_* (middle, 0.38 ± 0.01 in control, 0.29 ± 0.03 in 3% Iso, *n* = 7, *p* = 0.009, paired-sample *t* test), and *q* (bottom, 40.0 ± 0.2 pA in control, 42.0 ± 0.5 pA in Iso, *n* = 7, *p* = 0.572, paired-sample *t* test). **p < 0.01, N.S., no significance.

The lack of isoflurane effect on mEPSC amplitude suggests that its site of action is presynaptic, as reported previously for this and other volatile anesthetics (Takenoshita and Takahashi, 1987; Kullmann et al., 1989; Wu et al., 2004; Baumgart et al., 2015). To identify quantal parameters involved in isoflurane action, we performed variance-mean analysis (Clements and Silver, 2000; Koike-Tani et al., 2008). EPSCs were evoked under different extracellular Ca^2+^/Mg^2+^ concentration ratios, and 4-AP(10 μM) was supplemented to maximally increase the release probability *p_r_* (Fig. 2*A*). The variance-mean plots of EPSC amplitudes, made before and 10 min after isoflurane application, provided values of quantal parameters, *N*, *p_r_*, and *q* from parabola fit curves (see Materials and Methods). The quantal size *q*, measured from the initial slope of the parabola, was unchanged after application of isoflurane (Fig. 2*B*), in agreement with unchanged mean amplitude of mEPSCs (Fig. 1*C*). The number of functional release sites *N* was measured from the *x*-axis intercept (*Nq*) of the parabola, divided by *q*. The release probability *p_r_* was then calculated from the mean EPSC amplitude (*Np_r_q*) divided by *Nq*. These analyses indicated that isoflurane reduced *N* and *p_r_*, by 43% and 24%, respectively (Fig. 2*B*; *n* = 7, *p* = 0.005 and *p* = 0.009 for *N* and *p_r_* respectively, paired-sample *t* test). These results suggest that isoflurane attenuates transmitter release by inhibiting multiple presynaptic targets.

### Isoflurane inhibits presynaptic voltage-gated ion channels and APs

**Figure 3.**
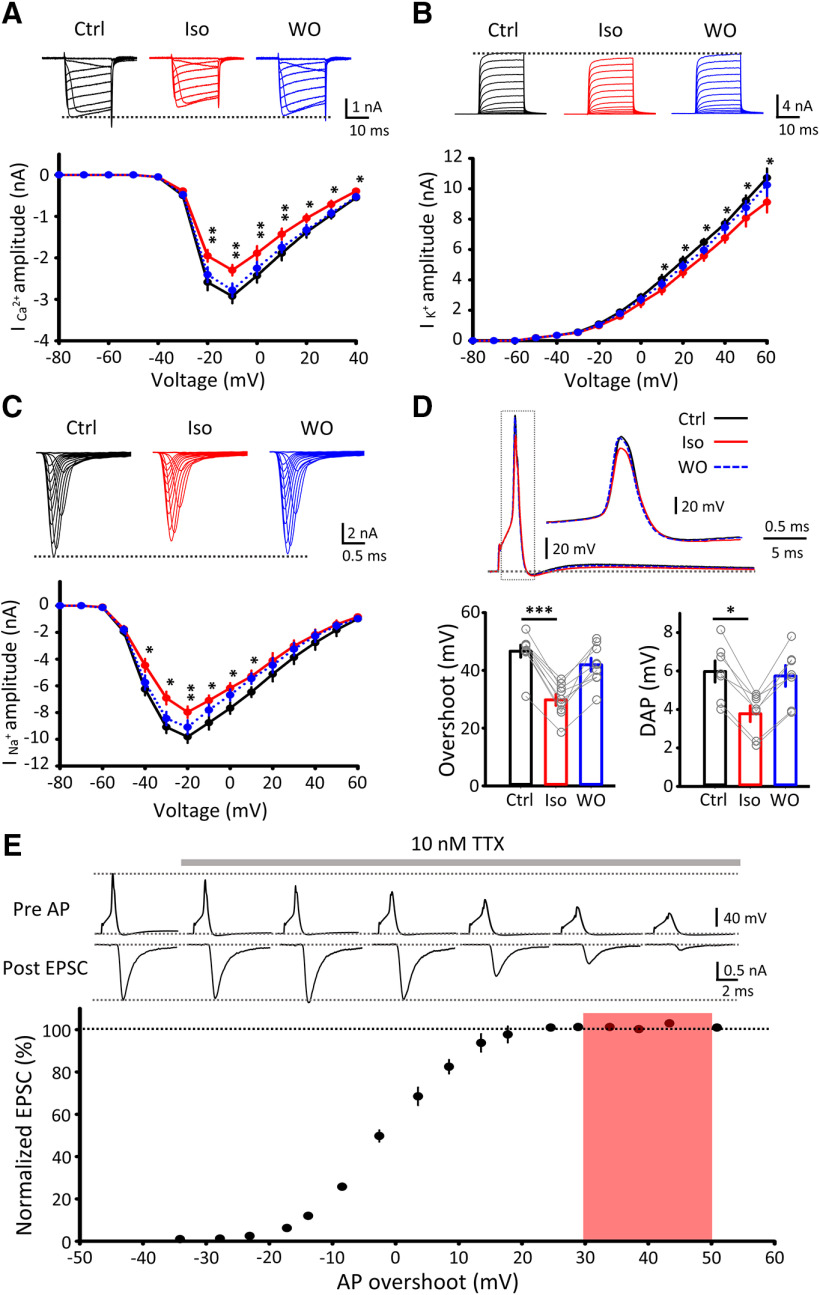
Isoflurane inhibits voltage-gated ion channel currents recorded from calyceal presynaptic terminals. ***A***, Top, Presynaptic Ca^2+^ currents before (Ctrl, black), 10 min after isoflurane application (Iso, red), and 10 min after isoflurane washout (WO, blue). ***A–C***, Dashed lines indicate maximal current amplitudes in controls. Bottom, Ca^2+^ current–voltage relationships in Ctrl (black), 3% Iso (red), and WO (blue). Peak amplitudes of presynaptic Ca^2+^ currents were significantly reduced by 3% Iso, between −20 mV and 40 mV (*n* = 8). ***B***, Top, Presynaptic K^+^ currents in Ctrl, 3% Iso, and WO. Bottom, K^+^ current–voltage relationships in Ctrl, 3% Iso, and WO. Isoflurane significantly decreased presynaptic K^+^ current amplitudes >10 mV (*n* = 6). ***C***, Top, Presynaptic Na^+^ currents in Ctrl, 3% Iso, and WO. Bottom, Na^+^ current–voltage relationships in Ctrl, 3% Iso, and WO. Isoflurane significantly decreased presynaptic Na^+^ current amplitudes between −40 mV and 10 mV (*n* = 4). ***D***, Top, Presynaptic APs in Ctrl, 3% Iso, and WO (superimposed). Bottom left bar graphs, Isoflurane significantly and reversibly reduced presynaptic AP overshoot (Ctrl 46.7 ± 0.9 mV, 3% Iso 30.6 ± 0.7 mV, WO 42.0 ± 0.9 mV, *n* = 9, *p* < 0.001, one-way repeated-measures ANOVA: *F*_(2,24)_ = 48.98, *p* < 0.001, top right bar graphs) but does not affect presynaptic AP half-width (Ctrl 0.28 ± 0.006 ms, Iso 0.29 ± 0.006 ms, WO 0.29 ± 0.004 ms, *n* = 9, one-way repeated-measures ANOVA: *F*_(2,24)_ = 0.73, *p* = 0.5) or RMP (Ctrl −69.63 ± 0.5 mV, Iso −68.85 ± 0.32 mV, WO −69.67 ± 0.31 mV, *n* = 9, one-way repeated-measures ANOVA: *F*_(2,24)_ = 1.28, *p* = 0.3). Bottom right bar graphs, Isoflurane also attenuated DAP (Ctrl 5.98 ± 0.55 mV, Iso 3.78 ± 0.42 mV, WO 5.75 ± 0.55 mV, *n* = 9, one-way repeated-measures ANOVA: *F*_(2,24)_ = 17.83, *p* = 0.02). ***E***, Top, Simultaneous recordings of presynaptic APs and EPSCs sampled from different epochs after bath-application of 10 nm TTX. aCSF contained kynurenic acid (1 mM). Bottom, The relationship between presynaptic AP overshoot and normalized EPSC amplitude during TTX application. EPSC amplitude remained unchanged even if the AP overshoot was reduced to 30.1 ± 1.9 mV by isoflurane (red shadow). EPSC amplitude declined only after AP overshoot was reduced by TTX to <13.5 ± 0.3 mV. Data are derived from 5 pairs. *p < 0.05, **p < 0.01, ***p < 0.001.

Isoflurane inhibits somatic T, N, and L type Ca^2+^ channels (Study, 1994) or recombinant Ca^2+^ channels (Kamatchi et al., 1999). However, it is not known whether it inhibits presynaptic Ca^2+^ channels mediating transmitter release. To determine this, we evoked presynaptic Ca^2+^ currents (I_Ca_) using 20 ms square command pulses, stepped up from a holding potential (−80 mV) to different membrane potentials, after pharmacological block of Na^+^ and K^+^ conductance (Fig. 3*A–C*). At the calyx of Held of posthearing rats, P/Q type (Ca_v_ 2.1) VGCCs predominantly mediate transmitter release (Iwasaki and Takahashi, 1998), as at many other mammalian central synapses (Iwasaki et al., 2000). Isoflurane significantly and reversibly inhibited I_Ca_ between −20 mV and 40 mV, without affecting the current–voltage relationship (Fig. 3*A*). The maximal magnitude of inhibition was 26.7 ± 0.4% (*n* = 8), which was comparable with the inhibitory effect of a metabotropic glutamate receptor agonist on presynaptic I_Ca_ (24.3%, Takahashi et al., 1996).

Voltage-gated K^+^ channels in presynaptic terminals regulate transmitter release by counteracting Ca^2+^ entry (Katz and Miledi, 1969). The main K^+^ channel shaping presynaptic APs is K_V_3 at the calyx of Held (Ishikawa et al., 2003). After blocking Na^+^ conductance with TTX, we tested the effect of isoflurane on presynaptic K^+^ channel currents. As reported at neuro-hypophysial terminals (Ouyang and Hemmings, 2005), isoflurane reversibly inhibited K^+^ currents for the voltage >10 mV (by 14.1 ± 0.3%, at 60 mV, *n* = 6; Fig. 3*B*). This effect of isoflurane on K^+^ currents could potentially counteract the inhibitory effect of isoflurane on transmitter release (but see below).

Volatile anesthetics inhibit Na^+^ currents in squid axons (Haydon and Urban, 1983) and at neuro-hypophysial terminals (Ouyang and Hemmings, 2005), as well as recombinant Na^+^ channel currents (Rehberg et al., 1996; Sand et al., 2017). Isoflurane attenuates presynaptic AP amplitude at calyces of Held (Wu et al., 2004) and at neuro-hypophyseal terminals (Ouyang and Hemmings, 2005). We examined whether isoflurane inhibits presynaptic Na^+^ channels at the calyx of Held (Leão et al., 2005). To optimize voltage-clamp control, we reduced extracellular Na^+^ concentration to 5% and blocked both Ca^2+^ and K^+^ conductance (see Materials and Methods). Na^+^ currents showed a peak at −20 mV and decreased in a graded manner above or below this potential, indicating adequate voltage-clamp control. Isoflurane significantly attenuated Na^+^ currents in a reversible manner between −40 and 60 mV, with a maximal inhibition of 18.8 ± 0.3% at −20 mV (*n* = 4; Fig. 3*C*). Unlike the report for recombinant Na^+^ channels (Sand et al., 2017), isoflurane had no effect on the inactivation rate of presynaptic Na^+^ currents (Na^+^ current decay time constant, 0.22 ± 0.01 ms in controls, 0.21 ± 0.01 ms with isoflurane, *p* = 0.28 at −20 mV, paired-sample *t* test).

We next examined the effect of isoflurane on presynaptic APs. Isoflurane significantly reduced AP amplitude from 116 ± 2.0 mV (overshoot, 46.7 ± 0.9 mV) to 98.6 ± 2.0 mV (overshoot, 30.6 ± 0.71 mV, *n* = 9, *p* <0.001, one-way repeated-measures ANOVA) (Fig. 3*D*). This magnitude of inhibition (17 mV, 15% in AP amplitude) was greater than that previously reported at prehearing calyces of Held (Wu et al., 2004) (5.5 mV or 5%, from 106 to 100.5 mV). Isoflurane also significantly attenuated depolarizing after potential (DAP; Fig. 3*D*) (Borst et al., 1995; Kim et al., 2010). The inhibitory effect of isoflurane on voltage-gated K^+^ channels (Fig. 3*B*) had no apparent effect on the AP waveform. Unlike previously reported (hyperpolarization by 1.2 mV) (Wu et al., 2004), isoflurane had no significant effect on resting membrane potential (RMP) (−69.6 ± 0.5 mV in control, −69.9 ± 0.31 mV in Iso, *n* = 9, *p* = 0.3, one-way repeated-measures ANOVA), ruling out the involvement of voltage-independent K^+^ channels in the inhibitory effect of isoflurane on transmitter release at this synapse.

To examine how a reduction ofpresynaptic AP amplitude affects transmitter release, we performed simultaneous recording of presynaptic APs and EPSCs, and applied TTX at a low concentration (10 nm) to allow gradual decrease in AP amplitude (Hori and Takahashi, 2009). When TTX reduced presynaptic AP amplitude by 17 mV (to 30.1 ± 1.9 mV in overshoot), EPSC amplitude remained the same (Fig. 3*E*), indicating that presynaptic AP amplitude has a wide safety marginfor transmitter release (Hori and Takahashi, 2009). EPSCs started to diminish only when AP amplitude was reduced by >33 mV (i.e., AP overshoot decline <14 mV). Thus, isoflurane inhibits Na^+^ channels and reduces presynaptic AP amplitude, but neurotransmitter release is protected from this mechanism at the calyx of Held.

### Effects of isoflurane on exo-endocytosis and recycling of synaptic vesicles

**Figure 4.**
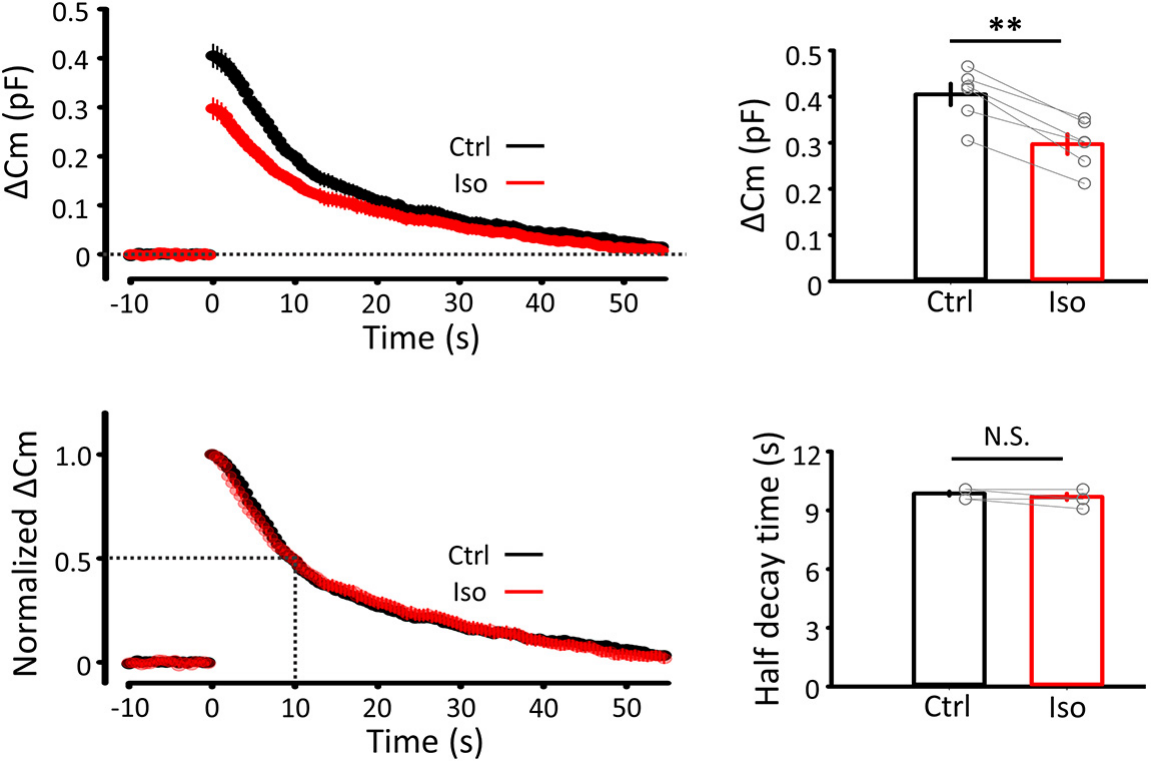
Isoflurane attenuated vesicle exocytosis without affecting endocytosis. Presynaptic membrane capacitance evoked by a 20 ms square pulse (stepped from −80 mV to 10 mV), with (red) or without (black) isoflurane (superimposed). Left top, Averaged traces (from 6 terminals) of exo-endocytic capacitance changes, with (Iso, red) or without (Ctrl, black) isoflurane (superimposed). Left bottom, ΔC_m_ traces normalized at the peak amplitude, with or without isoflurane (superimposed) showing no difference in endocytic time course. Right, Bar graphs represent inhibitory effect of 3% isoflurane on exocytic magnitude (top, ΔC_m_ from 0.41 ± 0.02 pF to 0.30 ± 0.02 pF, *n* = 8, *p* = 0.003, paired-sample *t* test) and no effect of isoflurane on endocytic time (bottom, half-decay time; Ctrl 9.86 ± 0.1 s, Iso 9.69 ± 0.17 s, *n* = 8, *p* = 0.175, paired-sample *t* test). **p < 0.01, N.S., no significance.

**Figure 5.**
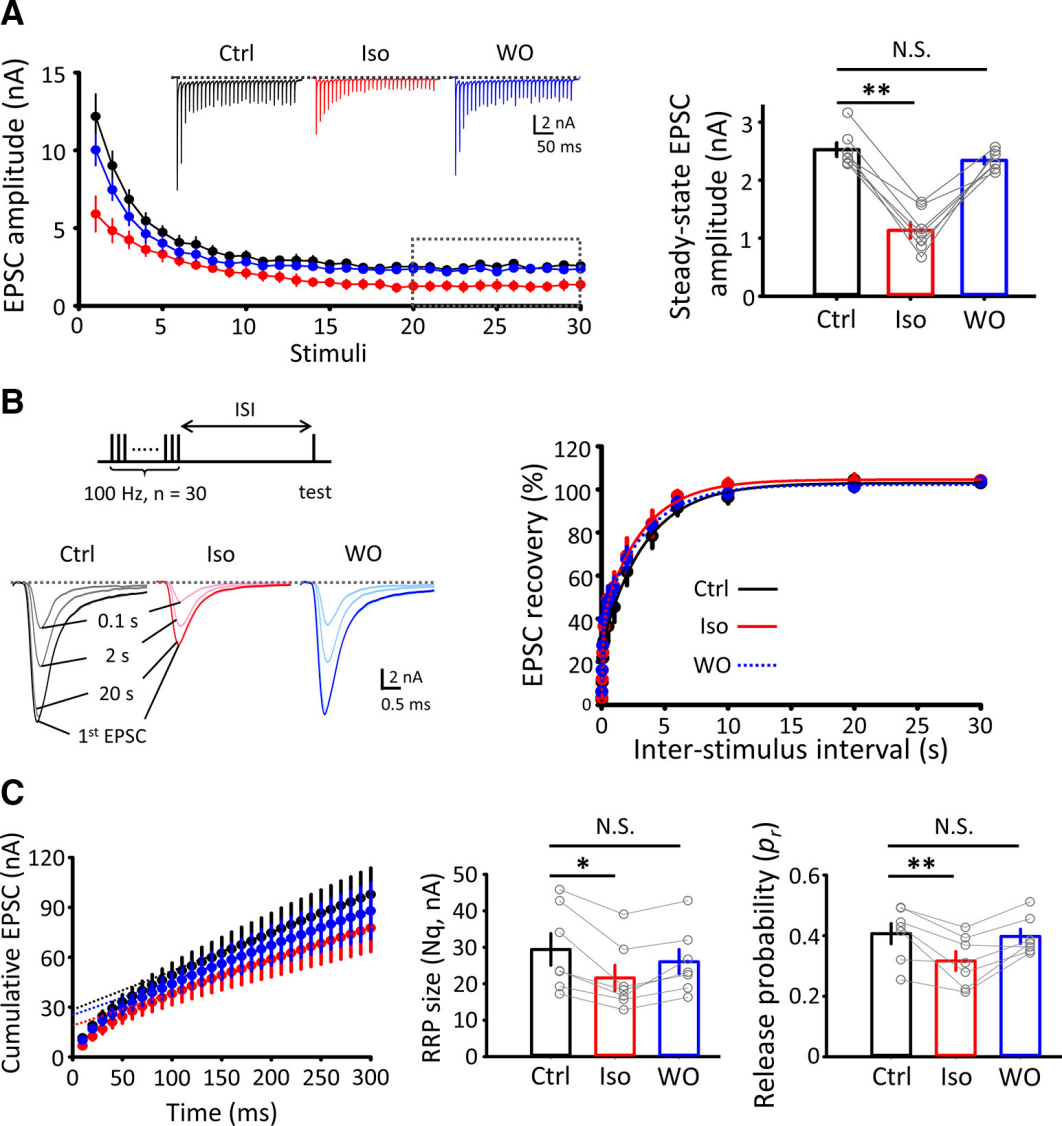
Isoflurane attenuated STD, but had no effect on the recovery of EPSCs from STD. ***A***, STD was induced by a 100 Hz train. Isoflurane (Iso, red) reversibly attenuated the magnitude of STD (right bar graphs) measured for 20-30 EPSCs (dashed line square). ***B***, Isoflurane had no effect on the recovery of EPSCs from STD (stimulation protocol on left top). Recovery time courses before (Ctrl, black), during isoflurane application (Iso, red), and after washout isoflurane (WO, blue). Right, Time course of recovery from STD before (black), during (red), and after washout (blue) of isoflurane. ***C***, Estimation of quantal parameters from cumulative EPSC amplitudes. Isoflurane reduced *Nq* (left bar graphs; Ctrl 29.42 ± 4.34 nA, Iso 21.58 ± 3.51 nA, WO 26.03 ± 3.4 nA, *n* = 7, one-way repeated-measures ANOVA: *F*_(2,19)_ = 13.15, *p* = 0.03) and *p_r_* (right bar graphs; Ctrl 0.41 ± 0.03, Iso 0.31 ± 0.03, WO 0.4 ± 0.02, *n* = 7, one-way repeated-measures ANOVA: *F*_(2,19)_ = 19.36, *p* = 0.008) in a reversible manner. *p < 0.05, **p < 0.01, N.S., no significance.

The inhibitory effect of isoflurane on *N* (Fig. 2) can be caused by direct inhibition of vesicle exocytosis, or indirectly by inhibiting vesicle recycling (Yamashita et al., 2005). To determine which mechanism underlies isoflurane action, we performed presynaptic membrane capacitance measurements from calyceal presynaptic terminals (Taschenberger et al., 2002; Sun et al., 2004; Yamashita et al., 2005, 2010; Eguchi et al., 2012). After blocking both Na^+^ and K^+^ conductance, exocytosis and subsequent endocytosis of synaptic vesicles were triggered by presynaptic I_Ca_ elicited by a square pulse, stepped from −80 mV to 10 mV for 20 ms. Isoflurane reduced the magnitude of exocytosis (ΔC_m_) by 29% (*n* = 8, *p* = 0.003, paired-sample *t* test) but had no effect on endocytosis (Fig. 4). These results of capacitance measurements at the calyx of Held are consistent with those reported from pHluorin experiments at cultured hippocampal synapses (Hemmings et al., 2005), indicating that vesicle endocytosis is not affected by isoflurane. In separate experiments of input fiber stimulation, during a train of 30 stimulations at 100 Hz, EPSCs underwent a short-term depression (STD; Fig. 5*A*). In addition to the initial EPSC amplitude, isoflurane significantly inhibited the steady-state amplitude of EPSCs during the train. The rate of recovery of EPSC amplitude from STD provides a measure of synaptic vesicle recycling (von Gersdorff et al., 1997). Isoflurane had no effect on the recovery time course of EPSC from STD (Fig. 5*B*). These results (Figs. 4, 5*B*) together indicate that isoflurane has no effect on vesicle endocytosis or recycling.

This stimulation protocol also provides estimates for quantal parameters from the cumulative amplitude histograms of EPSCs (Fig. 5*C*), as previously reported (Elmqvist and Quastel, 1965; Schneggenburger et al., 1999; Taschenberger et al., 2002). Isoflurane reduced *Nq* measured from 0-time axes of cumulative EPSC amplitude by 27% (from 29.42 ± 4.34 nA to 21.58 ± 3.51 nA, *n* = 7; *p* = 0.03, one-way repeated-measures ANOVA) and *p_r_*, calculated from the first EPSC amplitude divided by *Nq*, by 24% (from 0.41 ± 0.03-0.31 ± 0.03; *p* = 0.008, one-way repeated-measures ANOVA; Fig. 5*C*). These results are consistent with those from the mean-variance analysis (Fig. 2), confirming that isoflurane reduces the number of release sites and release probability.

**Figure 6.**
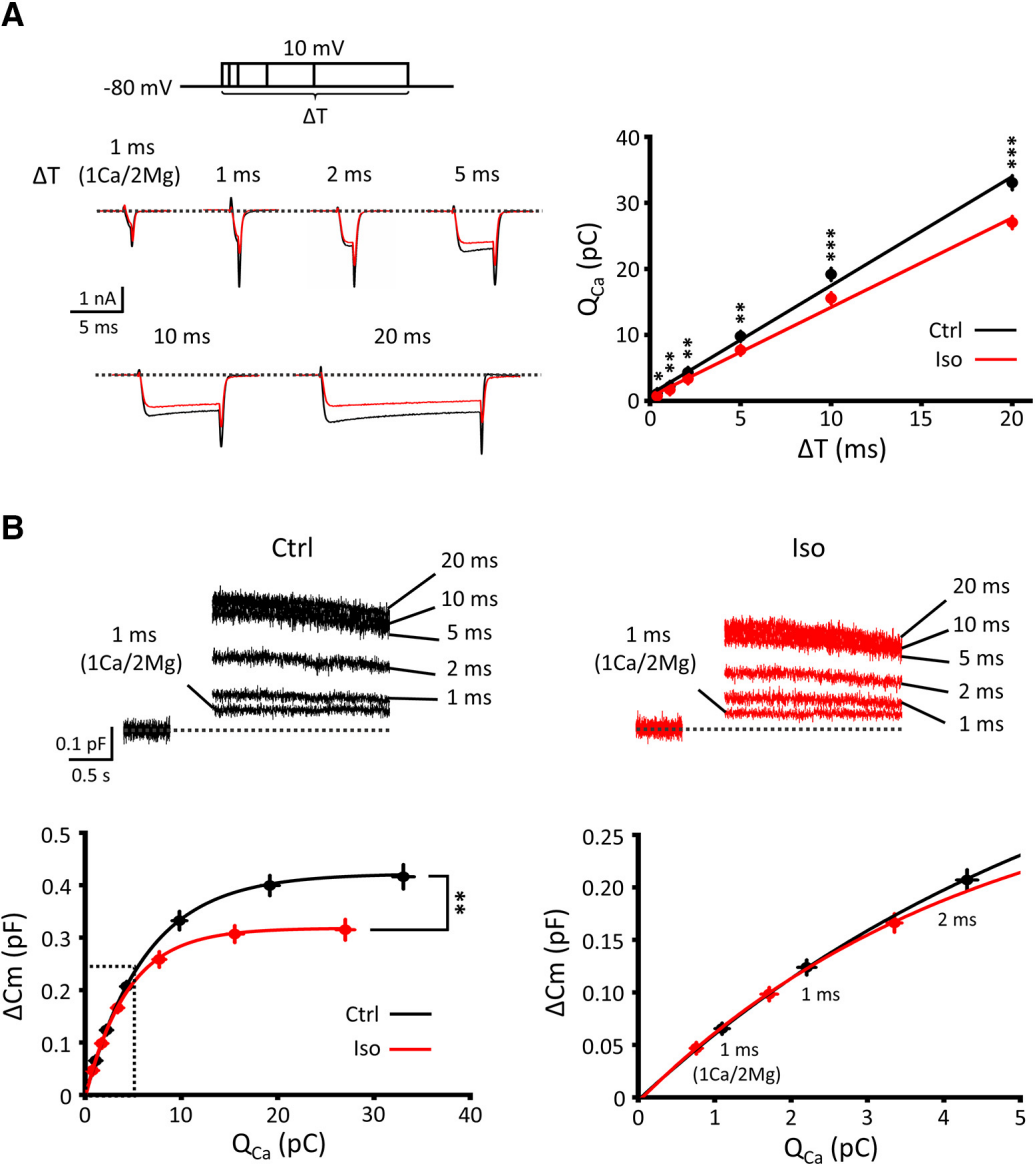
Isoflurane inhibits both presynaptic Ca^2+^ currents and exocytosis downstream of Ca^2+^ entry. ***A***, Left, Ca^2+^ currents evoked by square pulses of different durations (ΔT = 1, 2, 5, 10, and 20 ms) stepped from −80 mV to 10 mV (stimulation pulse protocol on top), in the presence (red) or absence (black) of isoflurane (superimposed). For 1 ms pulse stimulation, Ca^2+^/Mg^2+^ ratio in aCSF was either normal (2 mM/1 mM) or reduced (1 mM/2 mM). Right, Presynaptic Ca^2+^ charge transfer (Q_Ca_) at different pulse duration (Δ*T*), in the presence (red) or absence (black) of Iso (low Ca^2+^ data not included). Q_Ca_ in Iso was significantly less than that in control at all ΔT; *p* = 0.028 at 1 ms (1Ca/2 Mg); *p* = 0.004 at 1 ms (2Ca/1 Mg); *p* = 0.002 at 2 ms; and *p* < 0.001 at 5, 10, and 20 ms (paired-sample *t* test). ***B***, Top, ΔC_m_ induced by Ca^2+^ currents evoked by square pulses of different durations (superimposed), in the presence (Iso, red) or absence (Ctrl, black) of isoflurane. Bottom, Relationships between exocytic ΔC_m_ and presynaptic Q_Ca_, in the presence (Iso, red) or absence (Ctrl, black) of isoflurane. Bottom left, The maximal exocytic ΔC_m_ after isoflurane application (0.30 ± 0.02 pF) was significantly less than control (0.41 ± 0.02 pF, *p* = 0.003, paired-sample *t* test). Bottom right, ΔC_m_ for a low range of Q_Ca_ (left, dashed lines) showing an overlap of the Q_Ca_-ΔC_m_ relationships between control and isoflurane. *p < 0.05, **p < 0.01, ***p < 0.001.

We next investigated whether isoflurane might affect vesicle fusion machinery downstream of Ca^2+^ influx. To test this, we evoked exocytic membrane capacitance changes using presynaptic I_Ca_ elicited by square pulses of different durations (ΔT = 1, 2, 5, 10, and 20 ms), stepped up from –80 mV to 10 mV. Isoflurane significantly attenuated Ca^2+^ current charges (Q_Ca_) evoked by these pulses (Fig. 6*A*). Exocytic magnitude (ΔC_m_) represents the number of vesicles undergoing exocytosis at a time. When we increased command pulse duration, ΔC_m_ increased initially in proportion to Q_Ca_ but then reached a maximal level (Fig. 6*B*). Isoflurane reduced this maximal ΔC_m_ by ∼30%.

The Q_Ca_-ΔC_m_ relationships, with or without isoflurane, significantly overlapped at small Q_Ca_ range (<3 pC) induced by short depolarizing pulses (Fig. 6*B*), in agreement with a previous report on single AP-induced exocytosis (Baumgart et al., 2015). However, when more massive exocytosis was induced by longer depolarizing pulses, direct inhibition of exocytic machinery became a main mechanism of isoflurane action.

### Frequency-dependent inhibition of neurotransmission by isoflurane

**Figure 7.**
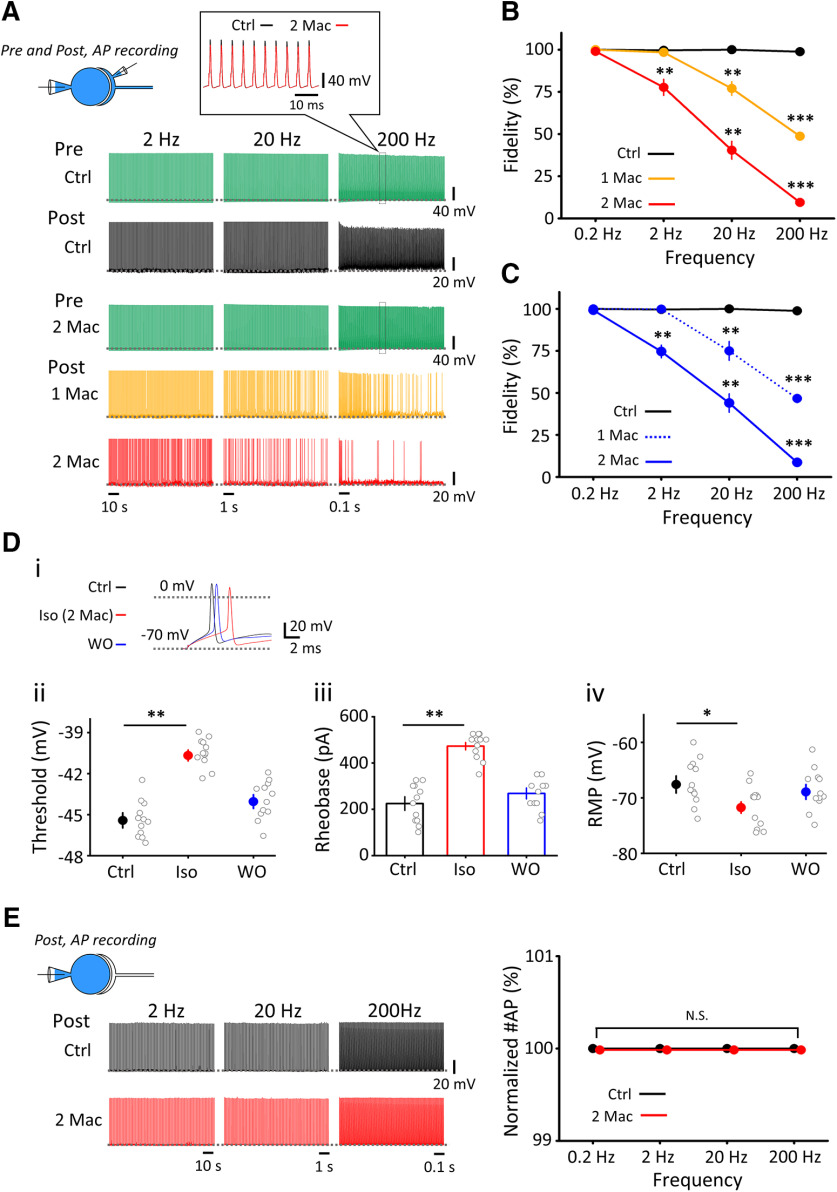
Frequency-dependent impairments of synaptic fidelity by isoflurane. ***A***, Simultaneous recording of APs from a presynaptic terminal and a postsynaptic neuron (recording mode illustrated on top left). Representative traces of presynaptic APs (green, control in the top row and with 2 MAC isoflurane on the third row, APs at 200 Hz shown at faster time scale in inset) and postsynaptic APs in control (black), 1 MAC isoflurane (1.5% Iso, yellow), and 2 MAC isoflurane (3% Iso, red). A total of 200 APs were generated by depolarizing pulses in presynaptic terminals at 0.2-200 Hz, and postsynaptic APs were recorded in whole-cell current-clamp mode at PT (31°C–33°C). Data at 0.2 Hz were not shown because of too long stretches (1000 s). ***B***, Fidelity (%) of synaptic transmission at different frequencies. Isoflurane at 1 MAC (yellow) or 2 MAC (red) impaired the fidelity of excitatory neurotransmission in a frequency-dependent manner. ***C***, Fidelity of synaptic transmission at different frequencies in the absence of bicuculline and strychnine in aCSF. Isoflurane was used at 1 MAC (blue, dashed line) or 2 MAC (blue line). **p < 0.01; ***p < 0.001; paired-sample *t* test. ***Di***, APs generated in a postsynaptic neuron before (black), during (red), and after washout (blue) of isoflurane (superimposed). ***Dii***, Isoflurane raised threshold of AP generation determined with dV/dt analysis. ***Diii***, Isoflurane increased rheobase current required for AP generation. ***Div***, RMP. Isoflurane hyperpolarized postsynaptic neuron. *p < 0.05; **p < 0.01; paired-sample t test. ***E***, Postsynaptic recording of APs (top). Left, The 200 APs evoked in a postsynaptic neuron by depolarizing current injections (1 ms, 1 nA) before (Ctrl, black) and 10 min after isoflurane application (3% Iso, red). Right, APs generated by 100% at different frequencies both in the absence (Ctrl, black) or presence of isoflurane (2 MAC, red). N.S., no significance.

Although isoflurane attenuates EPSC amplitude (Fig. 1*A*), spike transmission from a presynaptic terminal to a postsynaptic cell does not fail unless EPSPs diminish below the firing threshold. To clarify whether isoflurane affects spike transmission, we simultaneously recorded presynaptic and postsynaptic APs at the calyx of Held at near PT (31°C–33°C), at which synaptic depression is minimized (Kushmerick et al., 2006) and synaptic fidelity is improved (Piriya Ananda Babu et al., 2020). Although DAP reportedly supports presynaptic AP generation at high frequency (Kim et al., 2010) and isoflurane attenuated DAP (Fig. 3*D*), presynaptic APs did not fail during stimulation (200 APs), even in the presence of 3% (2 MAC) isoflurane at 200 Hz (Fig. 7*A*). In controls, postsynaptic APs followed presynaptic APs without a failure for the frequency range of 0.2–200 Hz (200 APs; Fig. 7*A–C*), indicating that the fidelity of transmission was 100%. Isoflurane (1.5–3%, 1–2 MAC) had no effect on the transmission fidelity at low-frequency ranges (<2 Hz at 1 MAC, and <0.2 Hz at 2 MAC, *n* = 5; Fig. 7*A*,*B*), with no AP failure. At higher frequencies, however, isoflurane reduced the fidelity of neurotransmission in dose-dependentand frequency-dependent manners (Fig. 7*B*). Since transmitter release evoked by high-frequency stimulation can be as massive as that evoked by long depolarizing pulse (Fig. 6*B*), direct inhibition of exocytic machineries, rather than inhibition of Ca^2+^ influx, likely underlies the isoflurane effect on high-frequency transmission.

MNTB neurons receive feedback inhibitory inputs from recurrent axon collaterals of neighboring neurons (Kuwabara et al., 1991; Kopp-Scheinpflug et al., 2008), but these inhibitory inputs are blocked by bicuculine and strychnine in the present experiments. Since volatile anesthetics prolong inhibitory postsynaptic responses (Nicoll, 1972; Mody et al., 1991), we tested whether inhibitory inputs might affect the effect of isoflurane on the fidelity of excitatory synaptic transmission in the absence of bicuculline and strychnine (Fig. 7*C*). The results were essentially the same, suggesting that inhibitory transmission has little influence on the fidelity of excitatory transmission at this synapse.

Might isoflurane cause AP failures also by a postsynaptic mechanism? We tested this possibility by evoking APs in postsynaptic MNTB neurons by direct injection of depolarizing currents (Fig. 7*D*). Isoflurane (3%) hyperpolarized MNTB neurons by 4 mV on average, increased firing threshold by 5 mV and accordingly increased rheobase currents by twofold (*n* = 12; Fig. 7*D*). However, isoflurane did not cause failure of postsynaptic APs evoked by trains of repetitive stimulations (200 stimuli at 0.2–200 Hz, *n* = 12; Fig. 7*E*).

### Effects of isoflurane on corticocortical spike transmission *in vivo*

**Figure 8.**
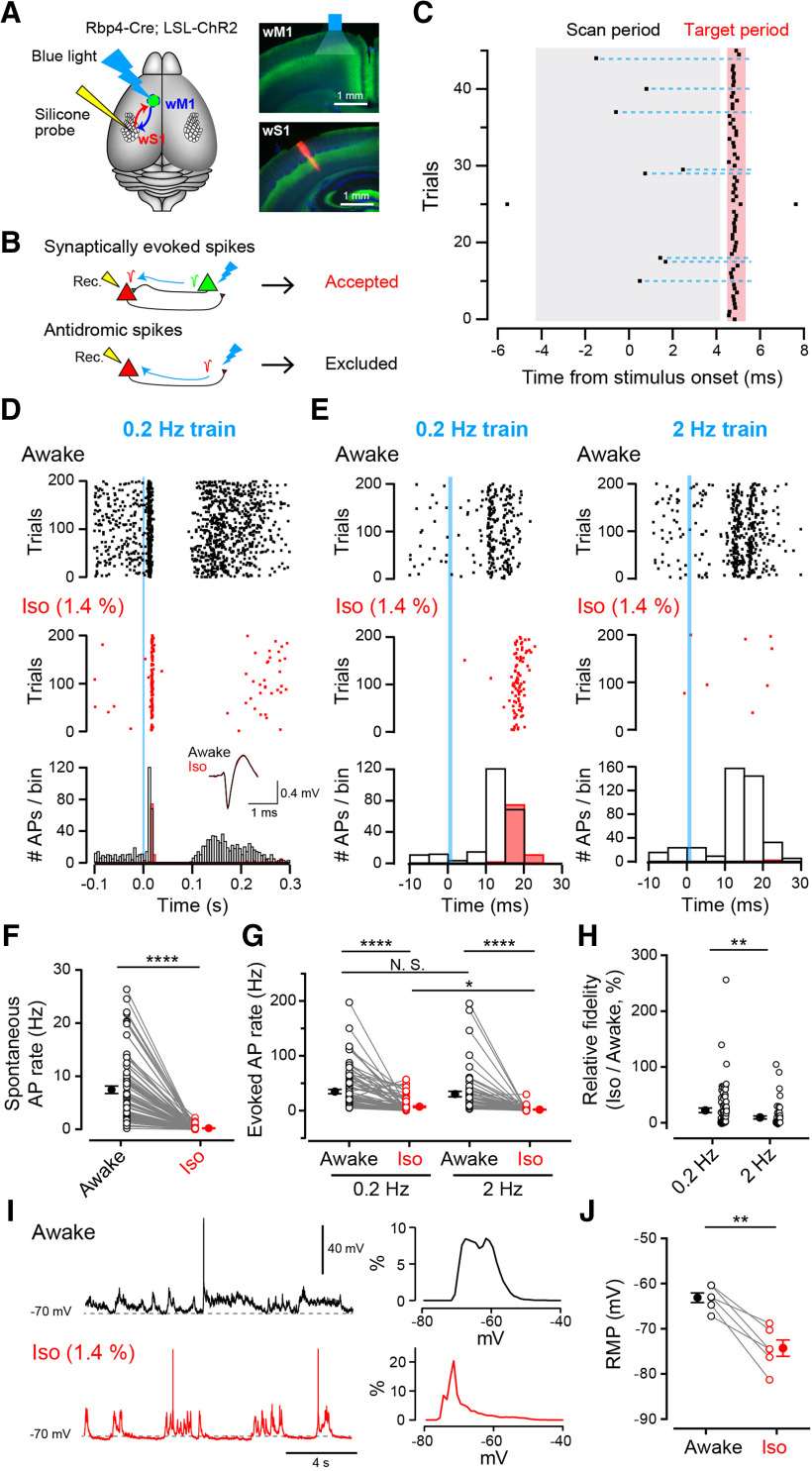
Inhibition of corticocortical excitatory synaptic transmission by isoflurane. ***A***, Left, Double-transgenic mouse line (Rbp4-Cre; LSL-ChR2) with L5-specific ChR2 expression was used. Presynaptic APs were evoked in wM1-L5 excitatory neurons by 1 ms blue light pulses, and postsynaptic APs were recorded in wS1 using a silicone probe. Right, Representative epifluorescent images of wM1 (top) and wS1 (bottom) regions. Green represents ChR2-eYFP; blue represents DAPI; red represents DiI (probe trace). ***B***, Selection of units for analysis. Units responding with APs at > 5 Hz at 5–25 ms after photo-stimulation onset are selected, whereas units showing antidromic spikes or unreliable AP generation from trial to trial were excluded. ***C***, Example raster plot from a unit with antidromic spikes. No spikes in the targeted time window (red shadow) are recorded in the presence of preceding spontaneous firings at the scan period (gray shadow), as indicated with dashed lines. ***D***, Representative recording of spikes before and after 0.2 Hz photo-stimulation (blue line). Raster plots in awake states (top, black) and after isoflurane inhalation (middle, red). Bottom, The number of APs per 5 ms bin is plotted. Averaged AP waveforms in awake states (black) and after isoflurane inhalation (red) were also shown in the inset (superimposed). ***E***, Representative recordings of spikes with 0.2 Hz (left) or 2 Hz (right) stimuli are shown in a shorter timescale. ***F***, Average frequencies of spontaneous APs before stimulation, in the absence (black bar) or presence (red bar) of isoflurane (1.4%). ****p < 0.0001 (Mann–Whitney *U* test). ***G***, Frequencies of APs evoked by photo-stimulation within 5-25 ms after stimulation onset, in the absence or presence of isoflurane. ****p < 0.0001; *p = 0.039; N.S., no significance; Dunn's multiple comparison test. ***H***, Fidelity of postsynaptic AP generation at 0.2 or 2 Hz in the presence of isoflurane relative to controls before isoflurane inhalation. **p = 0.0033 (Mann–Whitney *U* test). ***I***, Left, Example membrane potential recording from a wS1 neuron at the depth of 378 μm during wakefulness (top) and under isoflurane (1.4%) anesthesia (bottom). Right, Distributions of membrane potentials of the cell shown in the left panels. Data in awake (top) and isoflurane-anesthetized (bottom) states were obtained from a 30 s epoch for each. ***J***, Average RMPs of 6 neurons at L2/3 of wS1 were hyperpolarized by isoflurane inhalation. **p = 0.0013 (paired-sample *t* test). ***F***, ***G***, ***H***, ***J***, Open circles and gray lines represent individual units or cells. Filled circles with error bars represent mean ± SEM.

We next examined the effect of isoflurane on *in vivo* synaptic transmission at corticocortical excitatory synapses in awake head-restrained mice. The wM1 is anatomically and functionally connected to wS1 via mutual monosynaptic excitatory connections (Welker et al., 1988; Ferezou et al., 2007; Aronoff et al., 2010; Mao et al., 2011; Zagha et al., 2013; Sreenivasan et al., 2016). Using L5-specific ChR2-expressing mice, we evoked APs at 0.2 or 2 Hz in L5 pyramidal neurons in wM1 using optical stimulation (1 ms blue light pulses) (Fig. 8*A*). Spontaneous and evoked APs were recorded extracellularly from wS1 neurons using a 32-channel silicone probe. Blue light stimulation applied to the wM1 evoked APs in a subset of wS1 neurons within 5–25 ms after stimulation onset. When photo-stimulation excited wS1 axons projecting to wM1, a clear peak of antidromic spikes appeared 3–10 ms after stimulation, showing collisions with preceding spontaneous orthodromic APs (Fig. 8*B*,*C*). These units with antidromic spikes together with those showing unclear or unreliable increase in AP rate after photo-stimuli (data not shown) were excluded from further analysis to specifically interrogate the units showing synaptically evoked APs. The rate of spontaneous APs of the units was on average 7.4 ± 0.7 Hz (*n* = 83; Fig. 8*D–F*). The rate of APs evoked by photo-stimulation at 0.2 and 2 Hz was 35.0 ± 4.0 Hz (*n* = 83) and 30.3 ± 5.0 Hz (*n* = 60), respectively, after subtraction of spontaneous AP rate, with no significant difference between the stimulation frequencies (*p* = 0.23; Fig. 8*D*,*E*,*G*). Subsequently, AP firing ceased for ∼0.1s, presumably due to inhibition by internuncial neurons in wS1 (Mateo et al., 2011), until the next “rebound” firings occurred (Fig. 8*D*).

When isoflurane was inhaled at 1.4% (∼1 MAC), both spontaneous and evoked APs were markedly inhibited (Fig. 8*D–F*). After isoflurane inhalation, spontaneous AP frequency declined to 0.22 ± 0.04 Hz (2.9% of control before inhalation). The frequency of evoked APs also declined to 7.3 ± 1.4 Hz at 0.2 Hz stimulation, whereas it declined more markedly to 1.9 ± 0.6 Hz at 2 Hz stimulation (*p* = 0.039), indicating that isoflurane impaired the fidelity of postsynaptic AP generation to 22.2 ± 4.1% (*n* = 83) at 0.2 Hz, whereas more strongly to 9.5 ± 2.6% (*n* = 60) at 2 Hz stimulations (Fig. 8*G*). Thus, the inhibitory effect of isoflurane on synaptic transmission was significantly stronger at a higher frequency (*p* = 0.0039, Fig. 8*H*). These results are comparable with those at the calyx of Held in slice (Fig. 7), suggesting low-pass filtering effect of isoflurane on central synaptic transmission.

Since isoflurane hyperpolarized postsynaptic MNTB neurons (Fig. 7*E*), it may also affect the RMP of cortical neurons. We therefore performed whole-cell recordings *in vivo* from wS1 L2/3 neurons during wakefulness and under isoflurane anesthesia. The average RMPs during wakefulness were >−70 mV (−63.1 ± 1.1 mV, *n* = 6 cells) with spontaneous depolarizing responses occasionally generating APs (Fig. 8*I*,*J*). The membrane potential profile ranged broadly from the hyperpolarized “Down” state at ∼−70 mV to the depolarized “Up” state at ∼−55 mV (Fig. 8*I*,*J*). After isoflurane inhalation (1.4%), the average RMPs were hyperpolarized <−70 mV (−74.3 ± 1.8 mV, *n* = 6 cells), and the membrane potential profile shifted toward more negative potentials with reduced Up-state occurrences as reported previously after general anesthetics administration (Petersen et al., 2003). Such hyperpolarizing effects could underlie the decrease of spontaneous and evoked AP rates with isoflurane inhalation (Fig. 8*F*,*G*).

## Discussion

### Dual presynaptic targets of isoflurane

At the calyx of Held in rat brainstem slices, we have systematically addressed targets of isoflurane action. Clinical doses of isoflurane attenuated evoked EPSC amplitude without affecting the mean quantal size, measured from spontaneous mEPSCs or variance-mean analysis, indicating that the site of its action is presynaptic. Isoflurane significantly lowered the release probability (*p_r_*) and decreased the number of functional release sites (*N*). Presynaptic capacitance measurements revealed dual mechanisms underlying isoflurane action. Within a relatively low range of exocytosis, isoflurane reduces exocytosis via reducing Ca^2+^ influx without altering the Ca^2+^-exocytosis relationship as reported previously (Baumgart et al., 2015), whereas for greater exocytosis, isoflurane directly blocks exocytic machinery downstream of Ca^2+^ influx. The former and latter mechanism explains a reduction of *p_r_* and *N*, respectively, particularly since isoflurane had no effect on vesicle recycling. In agreement with inhibitory effect of isoflurane on release machinery, volatile anesthetics can reportedly bind to recombinant syntaxin (Nagele et al., 2005), and their inhibitory effects on neurosecretion can be eliminated by syntaxin mutant overexpressed in secretory cells (Herring et al., 2009).

Isoflurane reduced presynaptic AP amplitude at the calyx of Held as reported previously (Wu et al., 2004). However, low-dose TTX-application experiments in simultaneous presynaptic and postsynaptic recordings indicated that EPSC amplitude is protected from a reduction of presynaptic AP amplitude with a wide safety margin in such a way that EPSCs remain unaffected when AP amplitude is reduced by isoflurane. Such a safety margin is absent in voltage-clamp experiments, where EPSCs are evoked by AP-waveform command pulses (Wu et al., 2004; Hori and Takahashi, 2009). This is likely because of limited space-clamp control of an AP-waveform command pulse. Thus, a reduction of Na^+^ influx cannot be a mechanism for a reduction of transmitter release by isoflurane at the calyx of Held. However, this does not rule out the possibility that a reduction of AP amplitude might contribute to the inhibitory effect of isoflurane on transmitter release at other synapses having narrower safety margin or smaller presynaptic APs. Although isoflurane broadly inhibited presynaptic voltage-gated ion channels at the calyx of Held, only the inhibition of VGCCs could fully explained the inhibitory effect of isoflurane on transmitter release evoked by a single AP or a short depolarizing pulse.

### Frequency-dependent inhibitory effects of isoflurane on spike transmission in slice and *in vivo*

Excitatory neurotransmission is completed by a generation of postsynaptic AP. Even when transmitter release is reduced, as far as postsynaptic potentials (EPSPs) reach firing threshold, neurotransmission remains intact. In simultaneous presynaptic and postsynaptic recordings of AP trains, at the calyx of Held at near PT, isoflurane (1-2 MAC) had no effect on the initial or low-frequency transmission, but significantly inhibited high-frequency transmission in a frequency-dependent manner. Thus, a reduction of Ca^2+^ influx alone by isoflurane cannot inhibit spike transmission at this sensory relay synapse. In response to high-frequency inputs, EPSPs undergo STD, primarily due to a reduction in *N* (von Gersdorff et al., 1997; Schneggenburger et al., 2002). Isoflurane further decreased *N* by direct inhibition of exocytic machinery, thereby diminishing EPSPs below firing threshold. Therefore, with respect to the physiological excitatory transmission at the calyx of Held, the main target of isoflurane action is exocytic machinery rather than VGCCs. Unlike at the calyx of Held, however, at corticocortical synapses *in vivo*, isoflurane attenuated spike transmission evoked at low frequency (0.2 Hz), suggesting that the inhibition of VGCCs may also operate for the effect of isoflurane. Nevertheless, as at the calyx of Held, inhibitory effect of isoflurane on corticocortical spike transmission was much stronger at higher frequency (2 Hz). Thus, both at the calyx of Held and corticocortical synapses, exocytic machinery is likely an important target of isoflurane for attenuating high-frequency neurotransmission.

Together, the inhibitory nature of isoflurane on excitatory neurotransmission can be characterized as a low-pass filter. Weaker effect of anesthetics on low-frequency transmission seems favorable for the maintenance of life-supporting basal neurotransmission. Low-pass filtering effect of isoflurane is also consistent with large-scale slow-wave synchronization of cortical neurons during anesthesia (Mohajerani et al., 2010; Kuroki et al., 2018). Since high-frequency neuronal activity plays essential roles in the maintenance of consciousness (Herrmann et al., 2004; Lachaux et al., 2012), cognition (Sabatini and Regehr, 1999; Buzsáki and Draguhn, 2004; Uhlhaas and Singer, 2010), and motor-control (Sugihara et al., 1993), selective inhibition by volatile anesthetics of high-frequency transmission will effectively attenuate such integral neuronal functions, with minimal inhibition of basal neuronal functions.
